# Fungal Exocellular (1-6)-β-d-glucan: Carboxymethylation, Characterization, and Antioxidant Activity

**DOI:** 10.3390/ijms20092337

**Published:** 2019-05-11

**Authors:** Thais Vanessa Theis, Vidiany Aparecida Queiroz Santos, Patrícia Appelt, Aneli M. Barbosa-Dekker, Vaclav Vetvicka, Robert F. H. Dekker, Mário A. A. Cunha

**Affiliations:** 1 Departamento de Química, Universidade Tecnológica Federal do Paraná (UTFPR), Via do Conhecimento, km 01, 85503-390 Pato Branco, Paraná, Brazil; thaisvtheis@gmail.com (T.V.T.); vidianyqueiroz@yahoo.com.br (V.A.Q.S.); 2 Departamento de Química, Universidade Tecnológica Federal do Paraná (UTFPR), Rua Rosalina Maria Ferreira, 1233, 87301-899 Campo Mourão, Paraná, Brazil; patriciaappelt18@gmail.com; 3 Centro de Ciências Exatas, Departamento de Química, Universidade Estadual de Londrina, Rodovia Celso Garcia Cid, PR 445 Km 380, 86057-970 Londrina, Paraná, Brazil; anelibarbosa@gmail.com; 4 Department of Pathology, University of Louisville, 511 S. Floyd St, Louisville, KY 40292, USA; Vaclav.vetvicka@louisville.edu; 5 Programa de Pós-Graduação em Engenharia Ambiental, Universidade Tecnológica Federal do Paraná (UTFPR), Avenida dos Pioneiros 3131, 86036-370 Londrina, Paraná, Brazil; xylanase@gmail.com

**Keywords:** bioactive macromolecules, biopolymer, carbohydrate, exopolysaccharide, lasiodiplodan

## Abstract

Exocellular (1→6)-β-d-glucan (lasiodiplodan) produced by the fungus *Lasiodiplodia theobromae* MMPI was derivatized by carboxymethylation using different concentrations of a derivatizing agent. Lasiodiplodan was derivatized by carboxymethylation in an attempt to increase its solubility and enhance its biological activities. Carboxymethylglucans with degrees of substitution (DS) of 0.32, 0.47, 0.51, 0.58, and 0.68 were produced and characterized. FTIR analysis showed a band of strong intensity at 1600 cm^−1^ and an absorption band at 1421 cm^−1^, resulting from asymmetric and symmetrical stretching vibrations, respectively, of the carboxymethyl group COO- in the carboxymethylated samples. Thermal analysis showed that native lasiodiplodan (LN) and carboxymethylated derivatives (LC) exhibited thermal stability up to 200–210 °C. X-ray diffractometry demonstrated that both native and carboxymethylated lasiodiplodan presented predominantly an amorphous nature. Scanning electron microscopy revealed that carboxymethylation promoted morphological changes in the biopolymer and increased porosity, and alveolar structures were observed along the surface. The introduction of carboxymethyl groups in the macromolecule promoted increased solubility and potentiated the hydroxyl radical-scavenging activity, suggesting a correlation between degree of substitution and antioxidant activity.

## 1. Introduction

Glucans are carbohydrate biopolymers found in the cell wall of cereals (oats and barley) and in microorganisms such as yeasts, filamentous fungi, and algae. These macromolecules belong to a heterogeneous group of glucose polymers, which consists of a linear chain composed of d-glucose units linked to each other through β-glucosidic bonds with or without branching [1]. Most commonly, the glucosidic bonds found in β-glucans are of the (1→3) type, but bonds of the (1→6) type, and those of mixed linkages, such as (1→3; 1→6) and (1→3; 1→4) are also found. These biomacromolecules are well known for their immunomodulatory ability, being considered as biological response modifiers (BRM) and aiding in the adaptation of cells to the environment against biological stresses [2].

The biological activity of β-glucans is influenced by different physicochemical parameters, including water solubility, molecular weight, primary structure, and branching. This diversity results in innumerable properties and enables a wide range of applications for such biomolecules in food, commercial biomedical, pharmaceutical, and cosmetic sectors [3].

Several scientific reports published in recent years have demonstrated that the biological functionalities of different polysaccharides have been relatively improved through the modification of their chemical structures [1,3,4]. The introduction of chemical groups into the macromolecule with the appropriate degree of substitution may influence the bioactivity of the glucans and, in this way, carboxymethylated, sulfonylated, acetylated, and phosphorylated derivatives have been obtained [3].

Among the chemical modifications of polysaccharides, carboxymethylation has been highlighted as a mechanism to improve its water solubility and the bioactive properties. Carboxymethylation consists of an etherification reaction that promotes the replacement of hydroxyls present in the monomeric units of glucose with carboxymethyl groups (CH_2_COO^−^) [5]. Besides increasing the solubility of the polysaccharides, carboxymethylation has contributed to the potentiation of antiviral and antitumor activities [6] and improved antioxidant activity [7] and antimicrobial activity [8].

Our research group identified the fungus *Lasiodiplodia theobromae* MMPI isolated from the tropical fruit pinha (sugar-apple, *Annona squamosa*) as a good producer of an exocellular (1→6)-β-d-glucan, named lasiodiplodan. Lasiodiplodan is a rare β-glucan, since this type of polysaccharide is not commonly found as an extracellular secretion (exopolysaccharide, EPS), but only as a component of the cellular wall. Commercial β-glucans have been extracted from the wall of yeast cells and mushrooms, but their extracellular production has many advantages in the down-stream processing steps, since this involves simpler operations such as precipitation of the biopolymer from cultivation broth with ethanol, and separation by centrifugation or filtration. Extraction from the microbial cell wall usually involves treatments with alkalis, cold and hot water, and alcohol-acid solutions that can promote modifications in the three-dimensional conformation of the macromolecule, and lead to the loss of bioactivities [9].

The main focus of the present study was the derivatization of lasiodiplodan by carboxymethylation, as well as the characterization of the carboxymethylated derivatives obtained. Considering numerous reports in the scientific literature demonstrating that the introduction of chemical groups within the polysaccharide macromolecule enhances or introduces new biological functionalities, we believe that the carboxymethylation of lasiodiplodan can be a strategic tool to obtain new biologically-active molecules.

## 2. Results and Discussion

**Figure 1:**
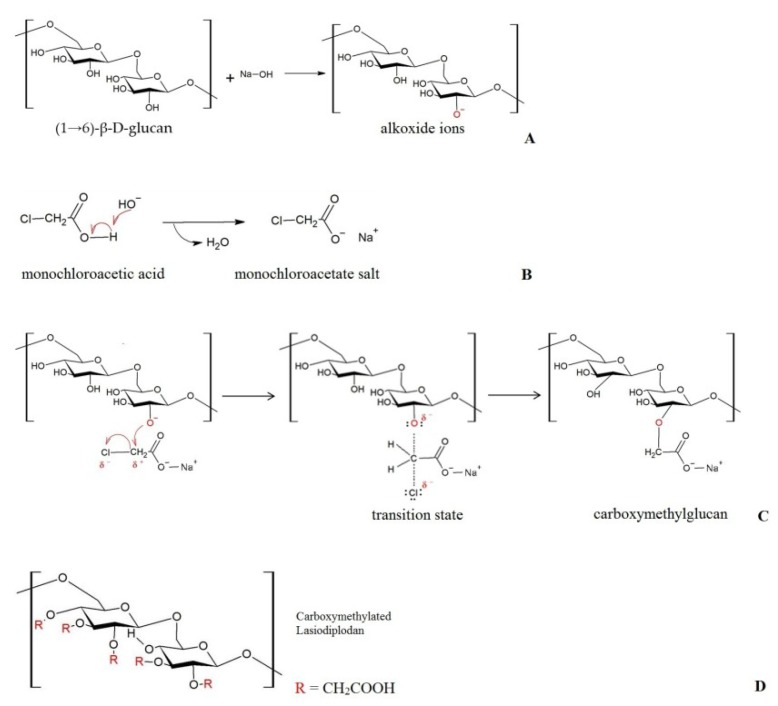
Illustrative representation of the reaction mechanism of carboxymethylation of lasiodiplodan. Formation of alkoxide ions (**A**), conversion of monochloroacetic acid to sodium monochloroacetate salt (**B**), nucleophilic bimolecular substitution (SN2) (**C**), and carboxymethylated lasiodiplodan (**D**). The positions shown for R indicate that the carboxymethylated group could be added at C-2, C-3, and C-4 positions on the glucose moieties.

The carboxymethylation reaction consists of an etherification reaction that aims at replacing the hydroxyls present in the monomeric glucose units with the introduction of carboxymethyl groups. In the reaction, alkoxide groups are formed, which react with an alkylating agent. Alkoxide groups are formed between sodium hydroxide and the hydrogens of the hydroxyl groups present in the polysaccharide macromolecule (Figure 1A). In alkaline medium, monochloroacetic acid is converted to the sodium monochloroacetate salt (Figure 1B). In the sequence, the carboxymethyl groups are formed through the Williamson synthesis by the nucleophilic bimolecular substitution reaction (SN2) between the formed alkoxide ions (nucleophilic agent) and the substrate (sodium monochloroacetate salt) (Figure 1C). Figure 1D depicts the structure of lasiodiplodan with all hydroxyl groups etherified with carboxymethyl groups.

### 2.1. Evaluation of the Degree of Carboxymethylation

The concentration of the derivatizing agent employed in the chemical modification protocol influenced the content of carboxymethyl groups inserted in the chemical structure of the macromolecule, lasiodiplodan. An increase of its concentration promoted the production of carboxymethyl-derivatives with higher degrees of substitution (DS) as can be seen in Table 1. Higher percentages of carboxymethyl groups (19.58% by mass) and DS (0.68) were verified when a mass ratio native lasiodiplodan (LN)/monochloroacetic acid of 1:11.5 was employed.

Carboxymethylated lasiodiplodan with a higher degree of substitution (DS 1.27) was obtained by Kagimura et al. [10] using a polysaccharide/derivatizing agent ratio (1:12) similar to the highest mass-ratio employed in the present study (carboxymethylated lasiodiplodan (LC)5, 1:11.5). However, it is important to note that these authors employed a higher reaction time (8 h) than that employed in the present study (4.5 h).

Several methodologies are found in the literature to obtain polysaccharides derivatized by carboxymethylation, which commonly use the same chemical agents (monochloroacetic acid and sodium hydroxide) but with different concentrations and reaction conditions. The preparation of carboxymethylglucans from different concentrations of the derivatizing agent has been widely reported. Bai et al. [11] obtained derivatives of β-glucan extracted from the cell wall of *Saccharomyces cerevisiae*, with increasing carboxymethylation degrees (DS of 0.325, 0.449, 0.771, and 0.945) as a function of the concentration of the derivatizing agent used in the reaction. These authors used amounts of monochloroacetic acid between 2.25 and 10.05 g for 10 g of yeast glucan. Similarly, Wang et al. [12] described that the use of higher concentrations of derivatizing agent in the carboxymethylation of a polysaccharide extracted from leaves of *Cyclocary apaliurus*, which also promoted derivatives with higher degrees of substitution (0.025, 0.171 and 0.193). In this work the authors used concentrations of 1.0 g, 2.0 g, and 3.0 g of monochloroacetic acid to 0.5 g of polysaccharide.

Chemic.al modifications of polysaccharides are often related to changes in sample water solubility. The insertion of chemical groups in the polysaccharide hydroxyls may decrease intramolecular and intermolecular interactions, making the macromolecule more hydrophilic [13]. 

In fact, as observed in Table 1, carboxymethylation contributed to an increase in the solubility of lasiodiplodan. The derivative with the lowest degree of substitution (LC1: 0.32) in our work had a solubility 17 times higher compared to that of the unmodified (native) lasiodiplodan sample. The derivatives LC2 (DS 0.47), LC3 (DS 0.51), LC4 (DS 0.58), and LC5 (DS 0.68) also showed higher solubility compared to the unmodified sample. However, increasing the degree of carboxymethylation did not promote increased water solubility. Higher solubility was observed in the derivative with a lower degree of carboxymethylation (DS 0.32, 67.99% solubility).

The introduction of carboxymethyl groups into the macromolecule promotes structural changes that modify the macromolecule–water interaction, improving its hydrophilicity. Carboxymethylated polysaccharides have a new polyanionic character and the repulsion of charges cause a greater extension of the chain, consequently promoting an increase in hydrodynamic size [5]. Wang et al. [14] evaluated the water solubility of five carboxymethylated derivatives of pachyman, a (1→3)-β-d-glucan extracted from *Poria cocos* sclerotia. The derivatives presented different degrees of solubility due to the degree of substitution, and similar to the result found in the present study, no direct correlation between the increase of the substitution degree and the increase of the solubility was seen.

### 2.2. Fourier Transform Infrared (FTIR) Spectroscopy

**Figure 2:**
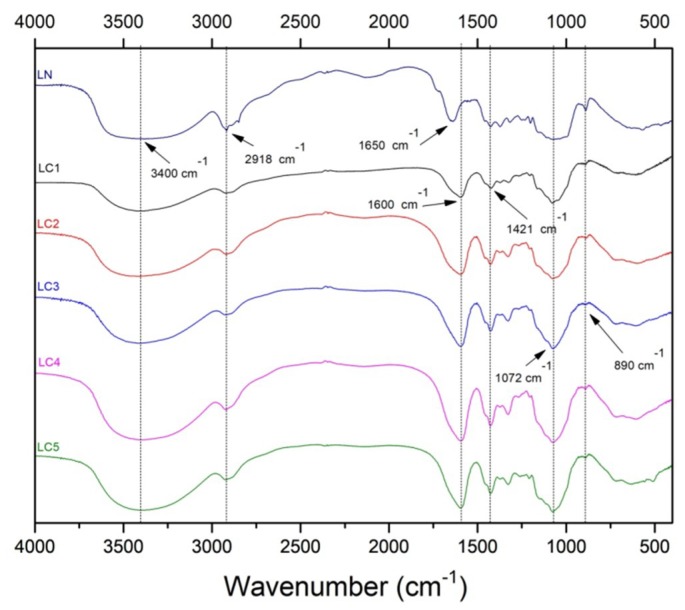
FTIR spectra of LN and LC1 (DS: 0.32), LC2 (DS: 0.47), LC3 (DS: 0.51), LC4 (DS: 0.58), and LC5 (DS: 0.68).

The FTIR spectra of LN and LC samples are shown in Figure 2. The wide band of strong intensity in the region between 3311 and 3424 cm^−1^ was observed in all spectra and is attributed to O–H stretch vibrations [15]. The absorption in 2918 cm^−1^ is due to sp3 C–H stretching [12,16]. The strong intensity band at 1600 cm^−1^ and the absorption band at 1421 cm^−1^ are the result of the asymmetric and symmetrical stretching vibrations of the COO^−^ group, respectively, and indicate the carboxymethylation of the biopolymer [12,17]. The weak band near 1421 cm^−1^ observed in the native lasiodiplodan (LN) sample has a peak near at 1419 cm^−1^, and may have originated from OH deformation (from a non-carboxymethylated group) and C–H bending [18,19]. 

The symmetrical and asymmetric stretching vibrations of the C–O–C bonds (characteristic sugar group) are attributed to bands of low intensity between 1246 and 1276 cm^−1^ (asymmetric vibrations) and of strong intensity between 1071 and 1078 cm^−1^ (symmetrical vibrations) [20]. The band at 1650 cm^−1^ verified in the sample LN is attributed to water adsorption [19]. It is noted that this band is overlapped by the asymmetric stretching vibration band of the COO^−^ group in the derivatized samples. The absorption in the 890 cm^−1^ region indicates the configuration of the β-type of lasiodiplodan that is weakened in the carboxymethylated derivatives [9,10,21]. 

### 2.3. Thermal Characterization

**Figure 3:**
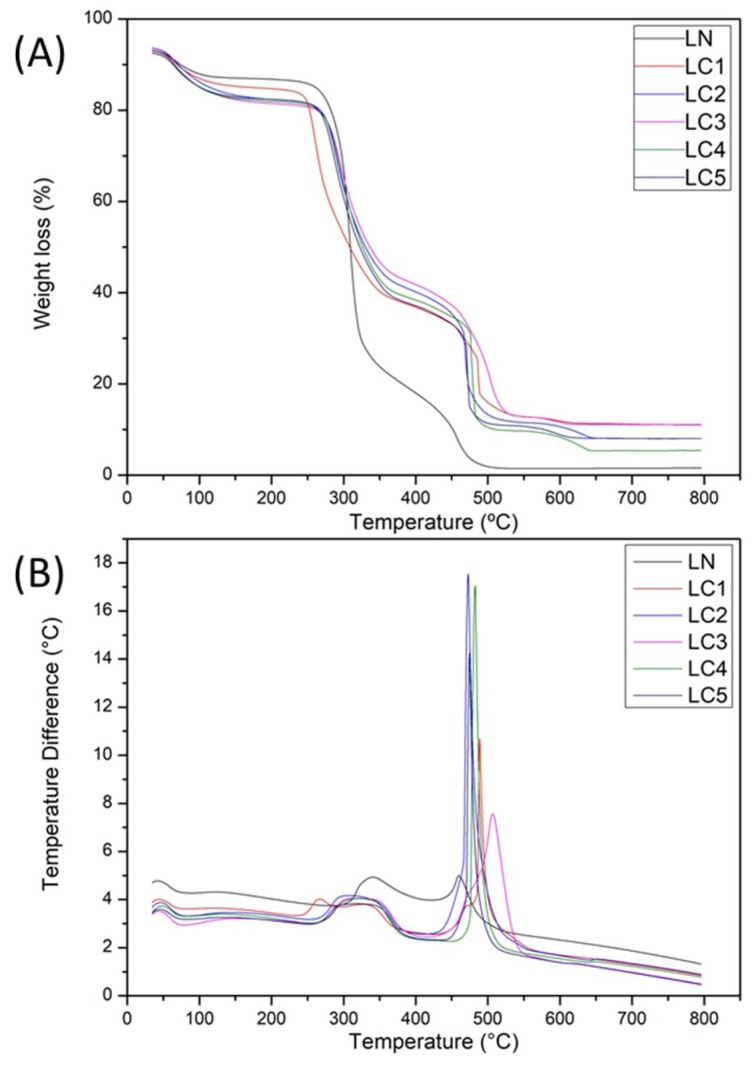
Thermogravimetric (**A**) and differential thermogravimetric (**B**) curves of LN and LC1 (DS: 0.32), LC2 (DS: 0.47), LC3 (DS: 0.51), LC4 (DS: 0.58), and LC5 (DS: 0.68).

Figure 3 shows the thermogravimetric (TGA) and differential thermogravimetric (DTA) analysis curves of LN and LC. The LN sample and the derivatives LC2 (DS: 0.47), LC3 (DS: 0.51), LC4 (0.58), and LC5 (0.68) presented four stages of mass loss, whereas the LC1 derivative (DS: 0.38) presented five stages. In the LN sample, the first stage of mass loss occurred up to 120 °C, being observed through of an exothermic peak at 41 °C by the DTA curve. Such loss of mass is attributed to the elimination of the water of hydration. A similar phenomenon was reported by Li et al. [22] in a starch sample, which described a first peak of loss of hydration water between 60 and 120 °C and a second peak corresponding to the degradation of the starch between 252 and 378 °C.

The second stage of mass loss in the LN sample occurred between 210 and 330 °C, being indicated by an endothermic peak at 290 °C and corresponding to the decomposition of the molecule. Two consecutive events with mass loss between 33 and 500 °C, indicated by two exothermic peaks in the DTA curve at 340 °C and 460 °C were identified. Possibly, the mass loss with exothermic peak observed at 340 °C corresponds to an oxidative degradation. Finally, the last stage of mass loss with an exothermic peak in the DTA curve at 460 °C corresponds to the final decomposition (carbonization) of the sample.

In the carboxymethylated derivatives, the first stage of mass loss occurred up to 160 °C. This mass loss is indicated by exothermic peaks in the DTA curve ranging from 45 to 48 °C and is attributed to water loss of the molecule. The second and third stages of mass loss in the sample LC1 (DS 0.32) occurred in a consecutive way between 200 and 400 °C and were indicated by two exothermic peaks (DTA) at 267 °C and 330 °C. The fourth and fifth events occurred between 400 and 530 °C, also consecutively, and were indicated by two exothermic peaks at 470 °C and 488 °C in the DTA curve.

The other derivatized samples (LC2, LC3, LC4, and LC5) showed a similar thermal behavior. The second stage of mass loss in these samples occurred between 200 and 400 °C and were indicated by exothermic peaks (DTA) ranging from 310 to 320 °C. The third stage occurred between 400 and 520 °C, indicated by exothermic peaks of the DTA curve that occurred from 473 to 507 °C, and was attributed to oxidative degradation of the samples. The fourth stage corresponding to the final decomposition of the derivatives was identified by endothermic peaks of the DTA curve that occurred from 610 to 640 °C, and was due to mass loss of the samples between 550 and 650 °C.

In the first stage of decomposition, referring to the loss of water of hydration, all the carboxymethylated samples (LC) showed higher mass loss compared to the native lasiodiplodan (LN). Such a phenomenon can be attributed to the reduction of hydrophobicity after the introduction of the carboxymethyl group by the chemical derivatization of the polysaccharide molecule. In fact, the increase of solubility of the derivatives in relation to the native sample was verified in the solubility evaluation assay. The differential thermogravimetric curves (DTA) of the unmodified (LN) and carboxymethylated (LC) samples also indicate that these compounds were more hydrophilic following carboxymethylation, since there was a small displacement (from 41 to 48 °C) from the values of the water-loss peaks in the DTA curves [23].

### 2.4. Morphological Characteristics of Native and Carboxymethylated Lasiodiplodan

**Figure 4:**
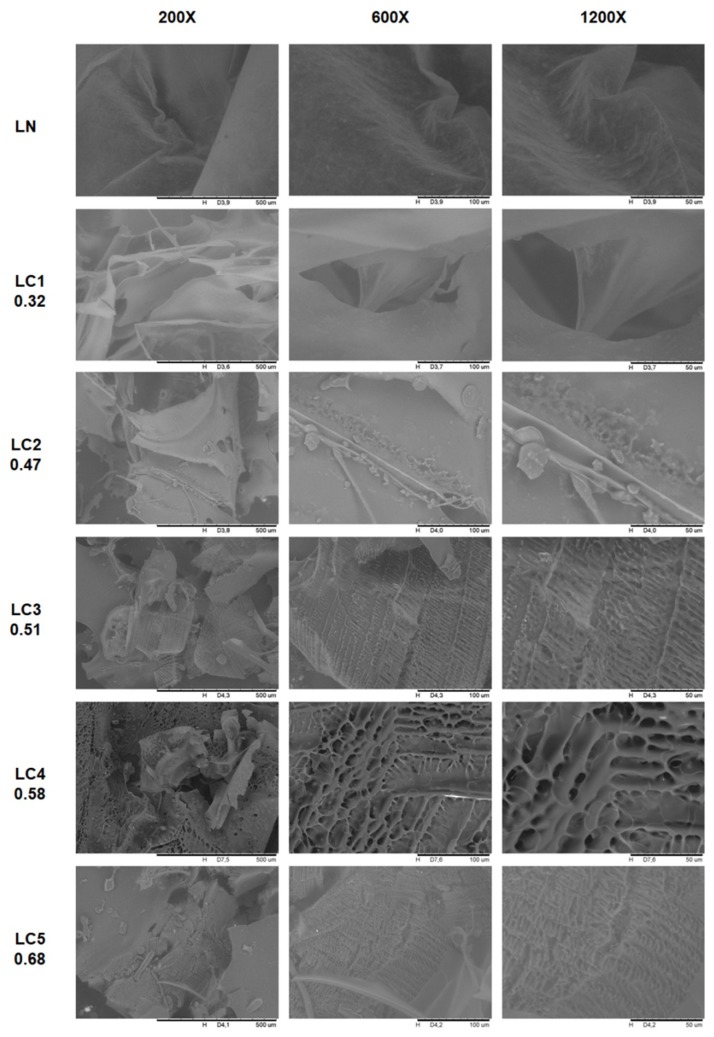
Micrographs (SEM) of LN and LC with different degrees of substitution 400X, 800X, and 1500X magnification.

The SEM micrographs of the LN and LC samples are shown in Figure 4. The LN sample presented a morphological structure, similar to thin films, was translucent, and had folds along its length. The derivatization by carboxymethylation promoted morphological changes on the surface of the biopolymer as seen in Figure 4. The image of the surface area of the LC1 sample shows less homogeneity than that of the unmodified sample (LN), with rips and orifices being observed along its surface. 

Lower surface uniformity was observed as the degree of substitution of biopolymer was increased. LC4 (DS: 0.58) and LC5 (DS: 0.68) derivatives showed the most heterogeneous surfaces among the analyzed samples. Regions with a certain porosity and presence of alveolar structures were seen most clearly in samples LC3 (DS: 0.47), LC4 (DS: 0.58), and LC5 (DS: 0.68).

Mittal et al. [24] evaluated the morphology of a galactomannan extracted from *Leucaena leucocephala* after carboxymethylation. The authors verified that the unmodified sample presented irregular granules on a smooth surface, and after the derivatization step, small pores that resulted in a superficial roughness were observed. Bhatia and Ahuja [25] observed that the carboxymethylation of arabinoxylan from *Psyllium* promoted morphological changes in the structure of the biopolymer. The fibrous and striated structure that was initially verified gave rise to a structure with a rough and porous surface.

### 2.5. X-ray Diffraction

**Figure 5:**
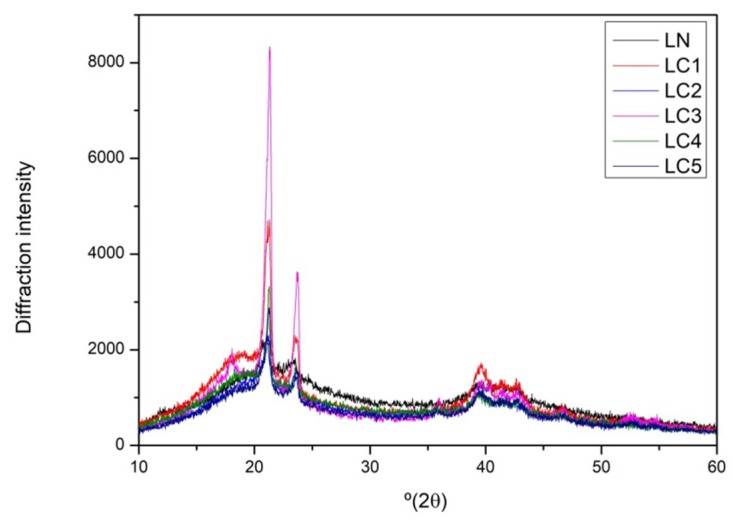
X-ray diffraction profiles of LN and LC1 (DS: 0.32), LC2 (DS: 0.47), LC3 (DS: 0.51), LC4 (DS: 0.58), and LC5 (DS: 0.68).

X-ray diffraction analysis is widely used to evaluate crystallinity, size, and orientation of crystallites [24]. The diffractographic profile of the LN and LC samples (Figure 5) shows that such biomaterials present an amorphous matrix, containing regions with certain molecular orientation. Three diffraction pronounced peaks at 2θ with values near to 21°, 23°, and 39° (2θ) were observed in the diffractograms of the LN and carboxymethylglucan samples obtained. The intensity of the peak observed at 21° (2θ) increased in the carboxymethylated samples, which is possibly associated with the introduction of a new functional group (carboxymethyl group) in this region after derivatization, and this may have contributed to a certain perturbation of the system.

Kagimura et al. [10] reported the occurrence of changes in the diffractographic profile in a sample of carboxymethylglucan (DS: 1.27) compared to native glucan. These authors observed the displacement and the disappearance of some peaks after carboxymethylation, and related these phenomena to the possible appearance of weakly crystalline regions with a non-preferential molecular orientation. However, they noted that although modifications were observed at specific points of the macromolecule after carboxymethylation, the main structure remained preserved and was similar to that found in the present study.

### 2.6. Antioxidant Ability

Reactive oxygen species (ROS), such as the superoxide ion (O_2_^−^), hydroxyl radical (OH^•^), and hydrogen peroxide (H_2_O_2_), are generated by natural metabolic processes or by exogenous factors [17,26]. The excessive accumulation of these species can cause several pathological effects, such as oxidative damage to DNA, proteins, and other macromolecules, as well as cellular degeneration [17,27]. Natural antioxidant compounds and foods containing high concentrations of antioxidants reduce oxidative damage and thus have an immunostimulatory effect [28].

The antioxidant activity of polysaccharides depends upon several structural parameters such as the degree of branching, molecular weight, monosaccharide composition, and presence of functional groups. The chemical modification of polysaccharides can alter several physico-chemical parameters, potentializing their antioxidant activity [28].

#### 2.6.1. Hydroxyl Radical Scavenging Activity (OH^•^)

**Figure 6:**
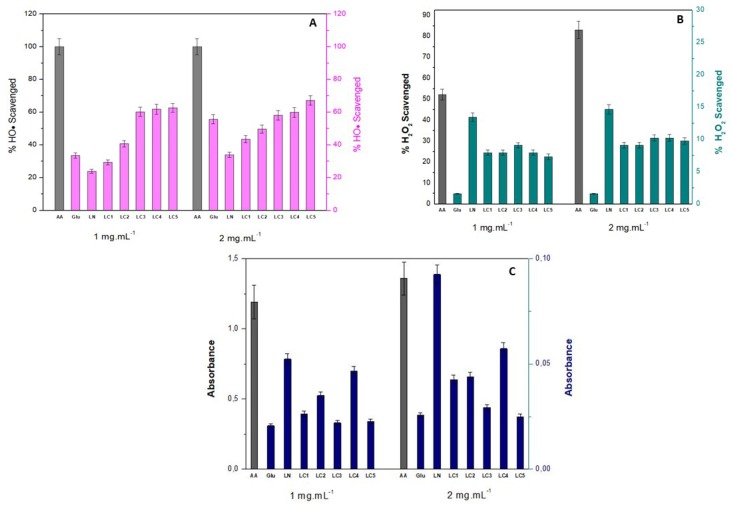
Antioxidant activity of native lasiodiplodan (LN), carboxymethylated lasiodiplodan (LC), glucose (GLU), and ascorbic acid (AA) (antioxidant standard) measured by (**A**) HO^•^ scavenging; (**B**) H_2_O_2_ scavenging, and (**C**) reducing power.

Most of the reactive hydroxyl radicals in biological systems are generated by the reaction between H_2_O_2_ and Fe^2+^, known as the Fenton reaction [29]. The hydroxyl radical exhibits extremely high reactivity and can promote severe damage to functional biomolecules in living cells. Antioxidant compounds are capable of sequestering and disrupting the radical reactions triggered by the OH^•^ radical [30]. According to Sheng and Sun [31], the hydroxyl radicals can be eliminated by antioxidant substances through three types of mechanisms: (i) electron transfer (OH+ + R → OH- + R+), (ii) hydrogen abstraction (RH + OH) → R^•^ + H_2_O), and (iii) addition to an aromatic ring or double bond breaking, which generate an addition product (OH^•^ + R = R → HO-R-R^•^). The ability of hydroxyl radical removal by LN and carboxymethylglucan (LC) samples is shown in Figure 6A.

Carboxymethylation of lasiodiplodan was demonstrated to enhance the ability to scavenge hydroxyl radicals. The hydroxyl radical-scavenging ability varied from 23.83% (LN) to 62.64% (LC5, DS: 0.68) at the concentration of 1.0 mg/mL, and when the concentration was increased to 2.0 mg/mL, the hydroxyl radical-scavenging activity ranged from 33.39% (LN) to 67.19% (LC5).

Comparing the hydroxyl radical-scavenging ability between the native sample (LN) and the sample with DS: 0.68 (LC5), 163% and 101% increases were observed at the concentrations of 1.0 mg/mL and 2.0 mg/mL, respectively. There seems to be a dose-dependent correlation between the glucan concentration (between 1.0 and 2.0 mg/mL) and hydroxyl radical-scavenging ability, especially in LN, LC1 (DS: 0.32), and LC2 (DS: 0.47) samples. On the other hand, native lasiodiplodan (LN) presented moderate hydroxyl radical-scavenging activity, both at the concentration 1.0 mg/mL (23.83%) and in the concentration of 2.0 mg/mL (33.39%). The antioxidant standard ascorbic acid, as expected, promoted 100% removal at both concentrations. The glucose monomer also demonstrated a low ability to remove the hydroxyl radical: 33.59% at the concentration of 1.0 mg/mL and 49.60% at 2.0 mg/mL.

Machová et al. [7] evaluated the ability of hydroxyl radical scavenging and the elimination of the (DPPH) 2,2-diphenyl-1-picrylhydrazyl radical by carboxymethylated derivatives of mannan, glucan, and dextran. These authors verified that the antioxidant activities of the derivatives were superior to those found in the native polysaccharide forms only when evaluated by the hydroxyl radical-scavenging method. They furthermore suggested that scavenging of the proton by the OH^•^ radical occurs preferentially on the carbon atoms of the pyranose ring, rather than at the hydroxyl groups. On the other hand, after the introduction of carboxymethyl groups, they observed increased antioxidant efficacy, which was attributed to the hydroxyl radicals being preferably eliminated by portions of the carboxymethyl group. It was speculated that the methylene hydrogens introduced in the macromolecule by the carboxymethyl group were more easily removed by the reactive hydroxyl radical species.

#### 2.6.2. Hydrogen Peroxide Removal Scavenging Ability (H_2_O_2_)

Although hydrogen peroxide (H_2_O_2_) is not very reactive, it is considered a major inducer of cell aging, attacking cellular energy production systems and playing an important role as an intermediary in the formation of reactive oxygen species (ROS) molecules when reacting with Fe^2+^ [17,32,33]. The potential for H_2_O_2_ scavenging by the LN and LC samples is shown in Figure 6B.

The antioxidant standard ascorbic acid showed a high ability to scavenge H_2_O_2_ at both the concentration of 1.0 mg/mL (52.2%) and 2.0 mg/mL (83.01%). On the other hand, the glucose monomer showed low H_2_O_2_ scavenging capacity at both concentrations (1.57%). Native lasiodiplodan was able to scavenge 13.41% of H_2_O_2_ content at the concentration of 1.0 mg/mL, and 14.63% at 2.0 mg/mL. However, the derivatization by carboxymethylation did not potentiate the ability for H_2_O_2_ scavenging as was observed in the HO^•^ scavenging assay. The H_2_O_2_ scavenging ability of the derivatized samples (LC) ranged from 7.31% (DS 0.51) to 9.09% (DS 0.68) at 1.0 mg/mL, and from 9.09% (DS 0.38 and 0.47) to 10.22% (DS 0.58 and 0.68) at the concentration of 2.0 mg/mL. No correlation was found between glucan concentration and degree of substitution in relation to the H_2_O_2_ scavenging capacity.

#### 2.6.3. Reducing Power

The reducing ability of a compound can be considered as an indicator of its potential antioxidant activity. In relation to the ferric ion (Fe^3+^), electron donation converts it into a non-reactive and more stable species, interrupting the formation and/or the free radical chain reaction [28]. In this assay, the compounds with antioxidant action convert the ferric ion (Fe^3+^) of ferric chloride into ferrous ion (Fe^2+^). The reaction of reduction of Fe^3+^ to Fe^2+^ is accompanied by a change in color from yellow to green, thus, the antioxidant potential can be measured by the intensity of the greenish coloration of the reaction mixture [34]. The reducing powers of LN and LC samples were determined by the reduction method using potassium ferricyanide. Higher absorbance values indicate greater reduction power, as shown in Figure 6C.

An increase of the concentration of native lasiodiplodan from 1.0 mg/mL to 2.0 mg/mL promoted a 77% increase in the reducing activity. On the other hand, as observed in the H_2_O_2_ scavenging activity, the derivatization by carboxymethylation did not potentiate the reducing power of derivatized lasiodiplodan.

The highest results of reducing power were observed in the LN (absorbance 0.092) and LC4 (absorbance 0.057) samples at the concentration of 2.0 mg/mL. The glucose monomer showed lower reducing power than that observed by native lasiodiplodan.

In a general context, the low reducing power of derivatized lasiodiplodan samples compared to the native polysaccharide may possibly be associated with a structural rigidity characteristic of the derivatized macromolecule, which could contribute to a lower exposure of active portions of the macromolecule [30]. The scientific literature generally supports that polysaccharides commonly exhibit relatively low reducing power.

Wang et al. [12] carboxymethylated a polysaccharide extracted from the *Cyclocarya paliurus* plant and evaluated its reducing power correlating to the degree of substitution of the modified molecules. In the mentioned work, the increase of DS led to a decrease in the reducing power of the samples, while the native polysaccharide demonstrated a greater reducing potential, which is similar to what we found in our work.

The introduction of substitution chemical groups into the polysaccharide molecules may contribute to the strengthening or weakening of the hydrogen bond dissociation energy. Therefore, the hydrogen donation capacity of modified polysaccharide derivatives may be increased or reduced, and as a consequence their antioxidant potential may be improved or decreased. The mechanisms of OH radicals and hydrogen peroxide scavenging, as well as the reducing antioxidant potential of the polysaccharides, are not well described and elucidated in the scientific literature. In our study, carboxymethylation contributed to the increase of the hydroxyl radical scavenging capacity, but this effect was not verified in relation to the capacity of scavenging of H_2_O_2_ and the increase of the reducing potential.

## 3. Materials and Methods

### 3.1. Biotechnological Process for Obtaining Lasiodiplodan

The production of lasiodiplodan (LN) was carried out by submerged fermentation in Erlenmeyer flasks (250 mL) containing 100 mL of nutrient media, comprising minimum salts medium [35], glucose (20 g/L), and 10 mL of standardized inoculum [36]. The initial pH of the medium was adjusted to 5.5 and the fermentation was carried out for 72 h at 28 °C under agitation conditions (150 rpm). The fermented broth was separated from the fungal mycelium by centrifugation (1500× g/20 min), and the exopolysaccharide precipitated with 3 volumes of absolute ethanol (4 °C, 12 h). The precipitate was then recovered by filtration and solubilized in distilled water under stirring and heating (60 °C), followed by exhaustive dialysis against distilled water (5 days) using MWCO dialysis tubes 12,000 Da, 1.3 in. (Sigma-Aldrich, St. Louis, MO, USA), and then lyophilized to give a white powder.

### 3.2. Carboxymethylation and the Degree of Substitution (DS)

Carboxymethylation of LN was performed following the protocol described by Wang et al. [14], with subtle adaptation. Lyophilized lasiodiplodan (1.5 g) was suspended in 65 mL of isopropanol with vigorous stirring for 15 min. Then, 25 mL of 20% NaOH (*w/v*) was added and kept under stirring for 3 h at room temperature. Subsequently, a solution of the carboxymethylating agent (mixture containing 2.5 g or 3.75 g or 5.0 g or 6.25 g or 8.75 g of monochloroacetic acid) mixed with 12.5 mL of 20% (*w/v*) NaOH and 31.5 mL of isopropanol was added slowly under stirring in order to obtain the carboxymethyl derivatives designated LC1, LC2, LC3, LC4, and LC5, respectively, with different DS. The reaction was run at room temperature for 3 h, and then the temperature was raised to 60 °C and maintained for 30 min. Next, a solution of the derivatizing agent solubilized in 12.5 mL of 20% NaOH (*w/v*) and 31.5 mL of isopropanol was again added, and the reaction mixture was maintained at 60 °C under stirring for 1 h. The reaction was stopped by cooling the mixture to room temperature and neutralizing it with a 1.5 mol/L hydrochloric acid solution to pH 7.0. Isopropanol was then removed from the mixture under reduced pressure in a rotary evaporator. The resulting solutions of carboxymethylated lasiodiplodan were dialyzed for 7 days against distilled water in dialysis tubes and lyophilized.

The degree of substitution (DS) of the derivatives was determined by the neutralization titration method, according to Tatongjai and Lumdubwong [37]. LC samples (150 mg) were dissolved in 100 mL of ultrapure water (Milli-Q) through vigorous homogenization for 3 min, and then centrifuged. The LC–Na salt present in the supernatant was converted to the acid form (LC–C–H) by passage through an ion-exchange column (6 cm × 1.5 cm) containing Amberlite IR-120 (H^+^) at a flow rate of 3 mL/min. After elution of the LC–C–H solution, the column was washed with 400 mL of ultrapure water (Milli-Q), and all of the eluted solution was collected (500 mL), and then the water was removed by lyophilization. The resulting lyophilized product was dissolved in 100 mL of distilled water and mixed with 3 drops of phenolphthalein, 2 mL of methanol, and 15 mL of 0.1 mol/L NaOH. The mixture was titrated with 0.1 mol/L HCl, and water was used as the blank. The DS was calculated from the following equations:

Wc = ([c × M_c_ × (V_b_ − V_s_) × 100])/m (1)

DS = ((W_c_ × M_a_))/(100 − W_c_) × M_c_ (2)

where Wc is the content of carboxymethyl groups in the sample solution (% by mass); c is the concentration of the HCl solution (0.1 mol/L) used in the titration; M_c_ is the molar mass of functional carboxymethyl groups that reacted with LN (58 g/mol); M_a_ is the molar mass of one anhydrous glucose unit (162 g/mol); V_b_ is the volume of HCl used for titrating the blank (mL), V_s_ is the volume of HCl used for titrating the sample (mL); m is the mass of the sample used in the titration of LC (mg); and DS is the degree of substitution of carboxymethyl groups in the polysaccharide samples.

### 3.3. Characterization of Native and Carboxymethylated Lasiodiplodan

#### 3.3.1. Evaluation of Water Solubility

The water solubility of LN and LC was assessed using the protocol describe by Kagimura et al. [10] with some modification. Samples of 10 mg were suspended in distilled water (10 mL), stirred for 24 h at 25 °C, and then centrifuged (8000× g, 15 min). Total sugars content of the supernatant was quantified by the phenol-sulfuric method [38] and directly related to the amount of soluble sample. The solubility in water was expressed as a percentage of soluble mass (% of soluble polysaccharide) in 100 mL of water (mg of sample/100 mL).

#### 3.3.2. Fourier Transform Infrared Spectroscopy (FTIR)

FTIR spectra of LN and the different LC samples were obtained using a FTIR Spectrometer Frontier (Perkin Elmer, Shelton, CT, USA) in the region of 4000–400 cm^−1^, with a 4 cm^−1^ resolution using the KBr disc method (ratio of sample/KBr of 1:100) and 32 accumulated scans. 

#### 3.3.3. Thermal Analysis

Lyophilized samples of LN and LC were submitted to differential thermal (DTA) and thermogravimetric analysis (TGA) on an SDT Q600 instrument (TA Instruments, New Castle, DE, USA). The mass loss was monitored between 30 and 800 °C in a synthetic air atmosphere with a flow rate of 50 mL/min and heating rate of 10 °C/min.

#### 3.3.4. Morphological Analysis by Scanning Electron Microscopy

Scanning electron microscopy (SEM) was used to analyze the surface morphology of the native polymer and its carboxymethyl derivatives. Micrographs were obtained on a benchtop Scanning Electron Microscope (TM3000) (Hitachi, Irving, TX, USA) using lyophilized samples. Samples were placed on carbon ribbons and images were taken at magnifications of 200, 600, and 1200×.

#### 3.3.5. X-ray Diffraction Analysis

X-ray diffraction patterns (XRD) of LN and LC samples were obtained using a Rigaku MiniFlex600 diffractometer, with copper lamp radiation source (CuKα = 1.5418 Å), 15 mA current, 40 kV tension, scanning range of 10 to 60° (2θ), 0.5°/min scanning speed, and a step width of 0.02° (2θ).

### 3.4. In-Vitro Antioxidant Activity

#### 3.4.1. Hydroxyl Radical Scavenging Activity Assay

Hydroxyl radical (HO^•^) scavenging activity was assessed according to Liu et al. [32] with subtle modification. Hydroxyl radicals were generated from FeSO_4_ and H_2_O_2_ and detected for their ability to hydroxylate salicylate. The reaction mixture (2 mL) contained 0.5 mL of FeSO_4_ (1.5 mmol/L), 0.35 mL of H_2_O_2_ (6 mmol/L), 0.15 mL of sodium salicylate (20 mmol/L), and 1 mL of the samples (containing 1 or 2 mg of polysaccharide). Ascorbic acid was used as a positive control. Absorbance of the hydroxylated salicylate complex was measured at 562 nm after an incubation period of 1 h at 37 °C. The percentage of hydroxyl radical scavenging was calculated as:

%OH scavenging = (1 − (A_1_ − A_2_))/A_0_ × 100 (3)

where A_1_ is the absorbance of the sample; A_0_ is the absorbance of the control (ascorbic acid), and A_2_ is the absorbance of the reagent blank without sodium salicylate.

#### 3.4.2. Hydrogen Peroxide Scavenging Activity Assay

The hydrogen peroxide (H_2_O_2_) scavenging capacity was measured according to Liu et al. [32]. The reaction mixture consisted of 0.5 mL of H_2_O_2_ (0.1 mmol/L, freshly prepared), 0.5 mL of sample suspension (1 or 2 mg/mL), 0.05 mL of ammonium molybdate (3% *w/v*), 5 mL of H_2_SO_4_ solution (2 mol/L), and 3.5 mL of KI solution (1.8 mol/L). The mixture was titrated with Na_2_S_2_O_3_ (5 mmol/L) until the disappearance of the yellow color. Scavenging activity was calculated as follows:

%H_2_O_2_ scavenging = (V_0_ − V_1_)/V_0_ × 100 (4)

where V_0_ is the volume of Na_2_S_2_O_3_ solution used to titrate the control mixture, and V_1_ is the volume titrated of the mixture containing the polysaccharide samples.

#### 3.4.3. Assay of Reducing Power

Reducing Power was assessed according to Liu et al. [32]. A mixture of 1 mL of lasiodiplodan sample, glucose, or ascorbic acid solutions (1 or 2 mg/mL) was incubated with 1 mL of potassium ferricyanide (1% *w/v*) at 50 °C for 20 min. The reaction was terminated by adding of 1 mL trichloroacetic acid (10% *w/v*) followed by the addition 2 mL of distilled water and 0.4 mL ferric chloride (0.1% *w/v*). Absorbance was measured at 700 nm. Higher absorbances of the mixture indicated greater reducing power of the sample. 

## 4. Conclusions

The analytical protocol used in the process of chemical derivatization of lasiodiplodan ((1-6)-β-d-glucan) was effective, promoting the production of carboxymethyl lasiodiplodan with different degrees of substitution as expected. An increase in the concentration of the derivatizing agent in the reaction of chemical modification led to derivatives with different degrees of substitution (DS: 0.32, 0.47, 0.51, 0.58, and 0.68). The introduction of carboxymethyl groups into the macromolecule led to a significant increase in solubility when compared to the unmodified polysaccharide. The derivative with the lowest DS (LC1: 0.32) presented a higher degree of solubility (67.99 mg/100 mL). On the other hand, the increase in the degree of carboxymethylation did not promote a subsequent increase in solubility. FTIR analysis confirmed that lasiodiplodan was carboxymethylated as revealed by specific bands of carboxyl groups. Native (LN) and derivatized (LC) lasiodiplodan presented relatively high thermal stability, up to temperatures between 200 and 210 °C, which is relevant considering the processing standards of the food and pharmaceutical industry sectors. Carboxymethylation promoted morphological changes in the superficial structure of the biopolymer, leading to the appearance of porosity and alveolar structures along the surface. Analysis by X-ray diffraction indicated that LN and LC have typical structures of amorphous biomaterials containing some crystalline regions. Regarding the antioxidant activity, the carboxymethylation potentiated the hydroxyl radical scavenging capacity by lasiodiplodan. However, it did not promote an increase in the H_2_O_2_ scavenging capacity, or an increase in the reducing power. The data obtained in this study demonstrated the possibility of obtaining derivatives with different degrees of carboxymethylation as a function of the concentration of the derivatizing agent used in the reaction mixture. Carboxymethylation of lasiodiplodan may contribute to enhanced biological functionalities and make this molecule more attractive for industrial applications in the food, pharmaceutical, chemical, and cosmetic sectors.

## Figures and Tables

**Table 1:** Degree of substitution (DS) and solubility of LN and LC samples.

Sample	Ratio of Lasiodiplodan/Monochloroacetic Acid (g/g)	Carboxymethyl Groups * (% by mass)	DS ^#^	Solubility ** (%)
LN	-	-	-	4.02 ^a^ ± 0.07
LC1	1:3	10.28	0.32	67.99 ^b^ ± 0.86
LC2	1:5	14.40	0.47	54.30 ^c^ ± 1.29
LC3	1:6.5	15.44	0.51	51.20 ^cd^ ± 1.92
LC4	1:8	17.19	0.58	49.58 ^d^ ± 0.39
LC5	1:11.5	19.58	0.68	45.50 ^e^ ± 0.66

* percentage of carboxymethyl groups introduced into the macromolecule, ^#^DS: Degree of substitution that corresponds to the average number of carboxymethyl groups introduced in each glucose monomer constituent of the polysaccharide. ** Solubility (%) = mg sample/100 mL. Equal letters in the same column indicate that there is no significant statistical difference (*p* < 0.05) at the significance level of 95%. LN: native lasiodiplodan, LC: carboxymethylated lasiodiplodan.

## Abbreviations

LN	Native lasiodiplodan
LC	Carboxymethylated lasiodiplodan
°C	Degree Celsius
θ	Theta
DS	Degree of substitution
